# Response: Commentary: Perceptual learning in autism: over-specificity and possible remedies

**DOI:** 10.3389/fnint.2016.00036

**Published:** 2016-11-09

**Authors:** Hila Harris, David Israeli, Nancy J. Minshew, Yoram S. Bonneh, David J. Heeger, Marlene Behrmann, Dov Sagi

**Affiliations:** ^1^Department of Neurobiology/Brain Research, Weizmann Institute of ScienceRehovot, Israel; ^2^Psychiatric Arrray, Kaplan Medical CenterRehovot, Israel; ^3^School of Medicine, Hebrew UniversityJerusalem, Israel; ^4^Center For Excellence in Autism Research, University of PittsburghPittsburgh, PA, USA; ^5^Department of Optometry, Bar-Ilan UniversityRamat-Gan, Israel; ^6^Department of Psychology and Center for Neural Science, New York UniversityNew York, NY, USA; ^7^Department of Psychology, Carnegie Mellon UniversityPittsburgh, PA, USA

**Keywords:** autism spectrum disorders, perceptual learning, generalization, psychophysics, adaptation, visual perception, repetition

In a recent study, we tested perceptual learning in adults with autism spectrum disorder (ASD) (Harris et al., 2015), employing the standard and well-established texture-learning paradigm [TDT; (Karni and Sagi, 1991; Sagi, 1995; Harris et al., 2012)]. In this paradigm, observers learn to discriminate an oriented texture target embedded at a fixed location in a background of elements having a different orientation. Performance is measured as a function of the time-interval between the onset of the target and a mask (stimulus onset asynchrony, SOA), with threshold defined as the minimal time (SOA) to reach a predefined criterion level of performance. Typical observers improve their performance (show reduced thresholds) with training across 3–4 days, but need to relearn the task when the target is moved to a different location in the visual field, showing specificity. We (Harris et al., 2015) reported similar results with observers with ASD, but unlike the typical observers who showed faster learning at the second location (Sagi, 2011), ASD observers showed difficulty in relearning the task at the second location, suggesting that the training with the target at the first location might have interfered with the training at the new, second location. We termed this anomalous poor learning “over-specificity” (OS) to reflect the narrowness of the learning and the failure to generalize, and quantified OS as the average threshold difference between the second and the first learning curves (for generalization OS < 0; specificity, OS = 0; over-specificity, OS > 0). A modified learning paradigm, where standard target trials were interleaved with no-target trials (“dummy” trials) during training, showed generalization of learning (OS < 0) across trained target locations, that is, non-specific, and generalizable learning, in both typical and ASD groups (Harris et al., 2015). When this newly defined OS measure is applied to previous studies with the experimental paradigm discussed here (Harris et al., 2012), and with other perceptual learning paradigms (Sagi, 2011), the results show mostly negative values, indicating some transfer of learning to the untrained stimulus. Perfect specificity (OS = 0) is rarely observed, and over-specificity (OS > 1) has never been observed before.

Mercado and coauthors (Mercado et al., 2016) have claimed that our results showing OS of learning in individuals with ASD, when using the standard training method (Harris et al., 2015), is explained by the higher initial thresholds of the ASD group relative to the other groups (standard typically developing controls (labeled TD); dummy ASD and dummy TD; Figure 2 in Harris et al. (2015). To evaluate the validity of this claim, here, we analyzed the dependence of OS on the initial threshold (measured on day 1), using measurements derived from each participant individually.

Figure 1 presents the individual OS values (the mean of the difference of the learning curves between the transferred new location and trained initial location from Figure 2 in Harris et al., 2015) as a function of the initial threshold. One can clearly see that OS for the ASD standard group does not depend on the initial threshold (*r* = 0.03; *p* = 0.94), while the other groups show negative correlations (*r* = −0.73, −0.58, −0.58; for the TD-standard, TD-dummy, and ASD-dummy groups respectively; *p* = 0.027, 0.077, 0.077 respectively; *p* < 0.001 for the three groups combined*)*. High initial thresholds can lead to both positive and negative OS, with TD participants showing negative OS (not positive as suggested by Mercado et al). The negative OS values are most probably a consequence of more learning (i.e., more improvement in performance with practice) when initial thresholds are high (Sagi, 2011). The positive OS observed with the ASD standard group is a novel result, not observed before in perceptual learning, and is not a direct consequence of higher initial thresholds, as claimed by Mercado et al. (2016).

**Figure 1:**
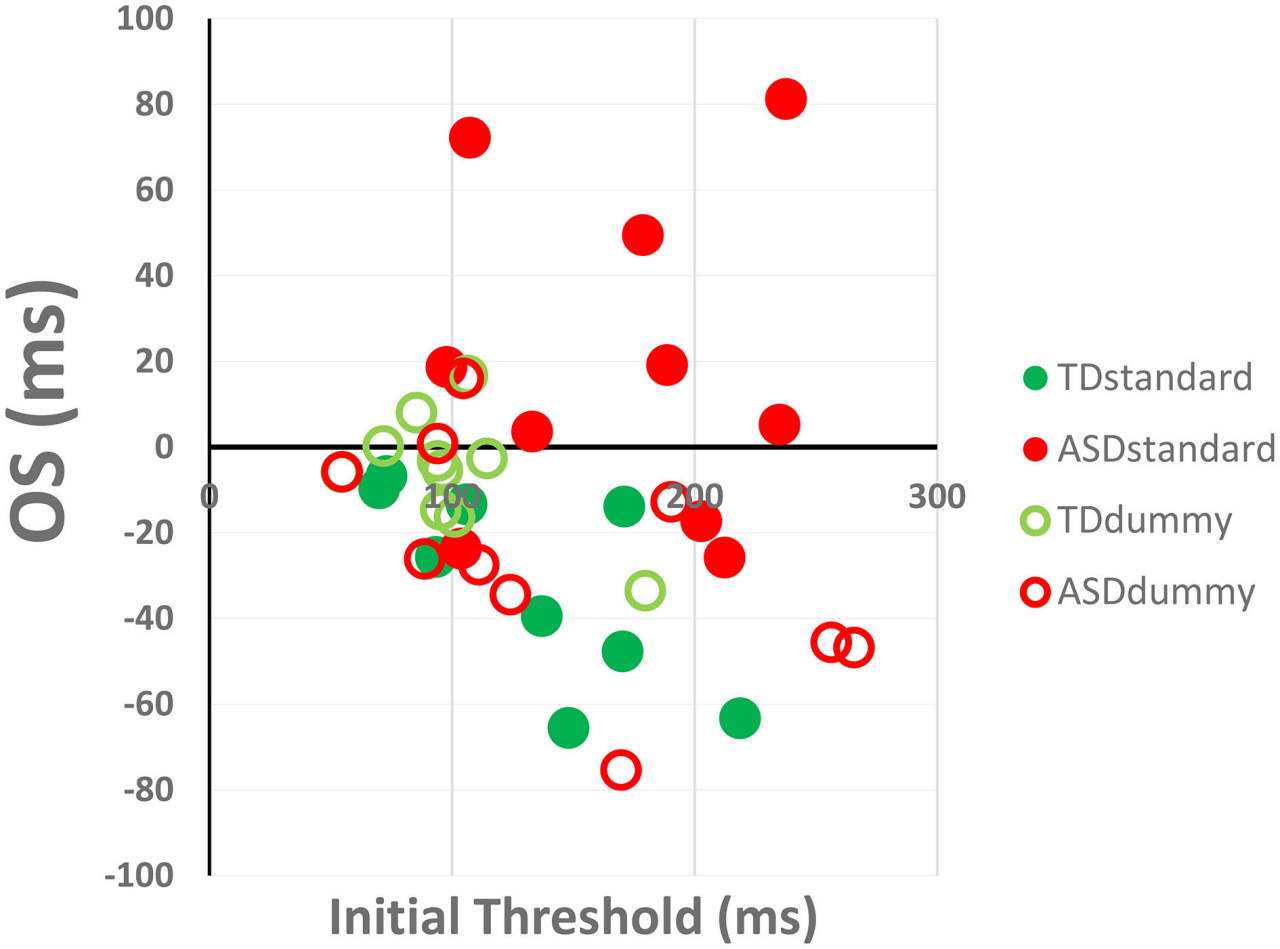
**Dependence of OS (Over Specificity) on the initial threshold measured on day 1**. OS is defined as the mean difference between the transferred and trained learning curves. A linear correlation shows no significant correlation for the ASD standard group (*r* = 0.03) but significant negative correlations for the other groups (*r* = −0.66, *p* < 0.001).

OS < 1 indicates better performance at the transfer location as compared with the trained location, and thus Figure 1 indicates better transfer for participants with higher initial thresholds. This result is typical in perceptual learning paradigms where the within-group threshold variance is larger in naïve participants relative to trained participants (Fahle and Henke-Fahle, 1996; Yehezkel et al., 2016). The outcome, then, is more learning when the initial threshold is high, often leading to higher transfer values, as expected from the reduced specificity of the initial phase of learning (Sagi, 2011).

Mercado et al. (2016) refer to our “dummy trials” as trials without targets. Our previous study (Harris et al., 2012) shows this interpretation not to be of general validity. Our results showed that the “dummy” effects depend on texture orientation and structure, in a way that cannot be explained by target presence or absence. For example, we found the dummy effects only when the background orientation was oriented 45° relative to target orientation, but not 90°.

Regarding pre-training, Mercado et al. (2016) refer to our findings (Harris and Sagi, 2015) showing dependence of the “dummy” effect on pre-training. This result is of no relevance here since the ASD participants generalized successfully in the “dummy” condition. Of course, the same pre-training procedure was applied to all groups.

In conclusion, the high Over-Specificity found for ASD participants trained with the standard method cannot be explained by higher initial thresholds, as suggested by Mercado et al. (2016). We are grateful for the opportunity to clarify our findings further and, in so doing, uncover novel findings (positive overspecificity values in ASD) that will prompt us to ask new questions and seek new answers.

## Author contributions

HH and DS analyzed data. DS, HH, MB, and DH wrote the manuscript. DI, NM, and YB reviewed the manuscript.

### Conflict of interest statement

The authors declare that the research was conducted in the absence of any commercial or financial relationships that could be construed as a potential conflict of interest.
